# Can Newly Acquired Healthy Behaviors Persist? An Analysis of Health Behavior Decay

**Published:** 2007-12-15

**Authors:** Ray M Merrill, Steven G Aldana, Roger L Greenlaw, Audrey Salberg, Hans A Diehl, Heike Englert

**Affiliations:** Department of Health Science, College of Health and Human Performance, Brigham Young University; Department of Exercise Sciences, College of Health and Human Performance, Brigham Young University, Provo, Utah; SwedishAmerican Center for Complementary Medicine, Rockford, Illinois; SwedishAmerican Center for Complementary Medicine, Rockford, Illinois; Lifestyle Medicine Institute, Loma Linda, California; University of Applied Sciences, Department of Nutrition, Muenster, Germany

## Abstract

**Introduction:**

We evaluated data from the Coronary Health Improvement Project (CHIP) to determine whether improved health behaviors associated with this intervention persisted or decayed during 18 months of follow-up.

**Methods:**

Participants were 348 volunteers aged 24 to 81 years from the Rockford, Illinois, metropolitan area enrolled in CHIP, a 4-week educational course delivered as lectures. The intervention taught the importance of making better lifestyle choices and improving dietary and physical activity behaviors. Physical activity and dietary behaviors were assessed at baseline, and changes in behaviors were assessed at 6 weeks and 18 months. Changes were evaluated according to quartile groupings of each variable at baseline.

**Results:**

No baseline differences were found between participants who dropped out and participants who provided data through 18 months. Mean changes significantly improved through 6 weeks for each of the 21 selected physical activity and dietary behavior variables except percentage of daily calories from carbohydrates. Mean changes significantly improved through 18 months for each of the 21 variables except calories from protein, alcohol, and whole grain servings. The percentage of participants who improved their physical or dietary behavior at 6 weeks ranged from 49% for percentage of daily calories from carbohydrates (64% at 18 months) to 91% for intake of dietary cholesterol per day (84% at 18 months). The level of change through 18 months for all variables was significantly influenced by quartile groupings at baseline. Physical activity improved significantly through 18 months only for participants in the lowest two quartiles of physical activity at baseline. Exercise decreased significantly through 18 months for participants in the highest quartile of physical activity at baseline.

**Conclusion:**

During an 18-month period, participants' physical activity and dietary behaviors improved significantly. Even though behavior improvement tended to be greater at 6 weeks, most healthy behaviors did not return to baseline levels after 18 months.

## Introduction

Benefits associated with a healthy diet, proper caloric intake, and participation in regular physical activity can be realized only if these healthy behaviors are maintained. When the healthy behaviors cease, the health benefits end (1,2). However, the adoption of new behaviors typically follows a predictable pattern. High adherence and dramatic behavior change characterize the initial days and weeks after an intervention, followed by a gradual return to previous behaviors over the ensuing months and years. This gradual migration away from newly adopted healthy behaviors toward former, less-healthy behaviors can be called *health behavior decay*.

A classic example of the concept of health behavior decay can be seen in the long-term weight loss literature (3,4). Many individuals attempting to use healthy behaviors to lose weight follow the same pattern. A period of initial weight loss is experienced when diet and exercise efforts are most consistent, followed by a period of health behavior decay, when new, healthy behaviors are replaced with former, less-healthy behaviors, resulting in weight gain. This pattern of health behavior decay can be seen in other interventions that have used healthy lifestyle change to lower high blood pressure (5,6), lower elevated blood lipids (7), and eliminate metabolic syndrome (8). Changes in nutrition and physical activity behaviors produce clinically significant improvements in risk, but with the passage of time the degree of risk reduction diminishes. Even a simple behavior such as taking a pill once each day can be difficult to maintain, as evidenced by the fact that less than half of individuals take their prescribed medications (9).

The amount of health behavior decay that occurs among intervention participants is largely determined by the characteristics of the intervention (e.g., design, length, intensity); level of individualization; and environmental factors, such as social support and barriers (e.g., time, money, lack of enjoyment). Well-designed, intensive interventions that have aggressive follow-up are assumed to be likely to result in less decay and, subsequently, lower risks long-term.

To acquire a better understanding of health behavior decay, we analyzed behavior change data from the Coronary Health Improvement Project (CHIP). CHIP was created to provide a lifestyle change program to the community to help reduce atherosclerosis-related diseases and improve the overall health of the public (10,11). The program, developed as a 30-day, 40-hour, live-lecture educational course, highlights the importance of making better nutrition, physical activity, and tobacco choices for preventing and reducing coronary heart disease. A clinical trial of CHIP showed dramatic improvements in nutrition and physical activity behaviors associated with clinically significant improvements in participants' insulin sensitivity, blood lipid levels, blood pressure, and body fat percentages (12). Most of the behavior changes and improvements in health risks among participants continued for 6 months (13).

In these trials, participants documented improvements in health knowledge, diet and physical activity behaviors, and changes in health risks (12,13). However, questions remain. Does health behavior decay exist? How long can significant improvements in behavior be maintained? Does decay occur equally in both nutrition and physical activity behaviors? The purpose of this study was to use the baseline, 6-week, 6-month, 12-month, and 18-month data from an ongoing CHIP study to obtain a better understanding of health behavior decay as it occurs in a lifestyle change program that is well-designed and intensive.

## Methods

### Subject recruitment and design

Participants were recruited by CHIP alumni groups, corporate clients, and the SwedishAmerican Medical Group; through targeted advertising by the SwedishAmerican Center for Complementary Medicine; and through marketing efforts by the Centers of Excellence. Complete recruitment details of the intervention have been published elsewhere (10). Recruitment efforts were aimed at adults aged 18 years or older in the greater Rockford, Illinois, metropolitan area. To be enrolled in the study, each participant had to be willing to participate in the intensive program and provide data at each of the five data collection periods. Eligible and interested participants provided informed consent, and participants were encouraged to participate with a spouse or significant other. The cost to participants was $290, which was returned if they completed the class. The Institutional Review Board of the SwedishAmerican Health System of Rockford, Illinois, approved the study on August 29, 2002. Participants included 348 volunteers aged 24 to 81 years from the Rockford, Illinois, metropolitan area.

### Intervention

The intervention for this study was CHIP (10). The primary objectives of CHIP were to improve participants' cognitive understanding of the importance of healthy lifestyles, nutrition and physical activity behaviors, and risk factors associated with diabetes, hypertension, cardiovascular disease, and cancer. Participants met for 4 weeks four times each week for 2 hours and received instruction through lecture-style presentations. Two classes of 174 participants each were held. Theory-based intervention planning was used to develop the curriculum, class design, alumni association (i.e., program designed to help participants maintain positive behavior changes), and take-home assignments (14-16). The intervention incorporated learning theory (i.e., behaviorism) in which changes in physical and dietary behaviors were promoted using health education and positive reinforcement from staff. The curriculum included the following topics: modern medicine and health myths, atherosclerosis, coronary risk factors, obesity, dietary fiber, dietary fat, diabetes, hypertension, cholesterol, exercise, osteoporosis, cancer, lifestyle and health, behavioral change, and self-worth. 

In conjunction with the CHIP lectures, participants received a health promotion textbook and workbooks that closely followed the discussion topics. The workbooks contained assignments with learning objectives for every topic and review questions to test participants' knowledge. These assignments were designed to help participants understand and integrate the concepts and information into their lives. Workbook assignments required approximately 30 minutes of participants' time, in addition to the time spent in each class session. Registered dietitians and medical professionals spoke to the group weekly, introducing participants to the latest nutritional and medical information related to the prevention of chronic diseases. Participants had access to scheduled shopping tours and cooking demonstrations given by a registered dietitian. The lecturer and program staff were present at each of the educational sessions and were available to answer questions about the presentations, workbook assignments, and program.

Participants were encouraged to follow preset dietary and exercise goals. The dietary goal involved adopting a more plant food–based diet that emphasized "as-grown" unrefined foods high in complex carbohydrates and fiber and low in fat, animal protein, sugar, and salt. Participants were encouraged to eat the following foods: whole grains, legumes, vegetables, and fresh fruits. The diet was composed of largely unrefined complex carbohydrates (65% to 70% of total calories); was low in fat (i.e., less than 20% of total daily caloric intake), animal protein, sugar, and salt; and was virtually free of cholesterol. Concurrently, program participants were encouraged to progressively work toward spending at least 30 minutes each day walking or performing some other exercise. Participants were given a pedometer and encouraged to keep an exercise log to record the miles walked each day.

At the completion of the program, participants were encouraged to join the Rockford CHIP Alumni Organization at an annual cost of $25 for individuals or $35 for couples. The purpose of the alumni organization was to help prevent relapse and help participants maintain their new behaviors. Alumni receive a monthly newsletter that contains news of health-promoting community events, such as healthy dinners, walking groups, and support-group meetings. The alumni were encouraged to attend special lectures on healthy living and ways to avoid relapse.

### Measures

Demographic data were collected at baseline. Attendance at each of the two classes was tracked. To assess dietary behavior, the Block 98.2 full-length dietary questionnaire was used (Block 98.2 Food Frequency Questionnaire, Block Dietary Data Systems, Berkeley, California). The Block 98.2 questionnaire has been extensively studied and validated (17-19). Data are self-reported, and the questionnaire is scanned optically and scored. The variables measured by this survey included but were not limited to the following: daily nutrients from food, percentage of calories from nutrients, fiber from different sources, and food-group servings per day.

To quantify physical activity behavior, a 7-day, self-recorded pedometer log was maintained by each participant. Participants wore the Walk4Life Model 2000 Life Stepper pedometer (Walk4Life, Plainfield, Illinois) on a belt at the right hip directly above the right knee cap each day for 7 days. Immediately before going to bed, participants recorded pedometer counts for the day and reset the number. Strike counts from pedometers are valid and reliable methods of monitoring and measuring free-living physical activity (20-22). One week before each data collection appointment, participants were contacted and reminded to keep track of their daily pedometer data for that week. Data were gathered by a registered nurse at baseline, 6 weeks, 6 months, 12 months, and 18 months.

### Statistical analysis

To ascertain whether any difference existed between participants who remained in the study through 18 months and those who dropped out, cross-tabulations were used to perform bivariate analyses between complete follow-up (yes vs no) and selected demographic variables, with statistical significance based on the *Χ* ^2^ test for equal proportions (23). Mean change scores in physical and dietary behavior variables were computed through 6 weeks and through 18 months. Mean change scores through 6 weeks and through 18 months were computed for physical and dietary behavior variables according to quartile groupings at baseline to isolate participants who already had healthy behaviors from those who did not. Analysis of variance (ANOVA) (24) was used to determine whether mean change scores differed significantly across the quartile groupings through 6 weeks and through 18 months for each of the physical activity and dietary behavior variables. Logistic regression (25) was used to evaluate whether improvements in the variables through 6 weeks and through 18 months were related to age, sex, race, marital status, annual family income, education, and employment status, after adjusting for quartile groupings at baseline. Confidence intervals (CIs) and statistical significance were based on an α level of .05. Because of the multiple comparisons in the final table of the results, the α level was adjusted to 0.00238 (.05/21) using a Bonferroni correction. The adjusted α level was used to determine significance of the demographic variables in the logistic regression models. Analyses were performed using SAS version 9.1 (SAS Institute Inc, Cary, North Carolina).

## Results

Of the 348 participants evaluated, none were lost to follow-up through 6 weeks, 45 were lost to follow-up through 6 months, 86 were lost to follow-up through 12 months, and 137 were lost to follow-up through 18 months. Hence, 211 (61%) were available for evaluation through 18 months. Participants attended 89% of the classes on average. After the intervention ended, 94 of the 211 participants joined the CHIP alumni association.

Bivariate analyses were used to compare participants who were available for evaluation through 18 months with those who were not, according to selected demographic variables (Table 1). The distribution across variables (i.e., sex, race, marital status, employment status, annual family income, and educational status) at baseline were not significantly different. Mean age for the two groups did not significantly differ at baseline (50 years for nondropouts vs 49 years for dropouts; *P* = .07) (data not shown).

Stepwise logistic regression was used to determine whether any of the physical activity or dietary behavior variables was associated with loss to follow-up through 18 months (Table 2). Only fruit servings was statistically significant (odds ratio, 1.42; 95% CI, 1.12-1.79) (data not shown).

Mean changes in selected physical activity and dietary behavior variables through 6 weeks and through 18 months are presented in Table 2. Mean changes improved substantially through 6 weeks for each of the physical activity and dietary behavior variables except carbohydrates. Mean changes improved substantially through 18 months for each of the variables except whole grain servings per day and percentage of calories from protein and alcohol. Although the percentage of participants who improved their physical or dietary behaviors at 6 weeks is more pronounced than at 18 months, the percentage who improved through 18 months remains high. For example, 63% of participants increased their physical activity, 42% decreased their alcohol consumption, and 84% decreased their intake of dietary cholesterol.

Mean changes in selected physical activity and dietary behavior variables through 6 weeks and through 18 months are also presented according to quartile groupings at baseline (Table 3). The level of change through 18 months for all variables was significantly influenced by quartile groupings at baseline. For example, significant improvements in physical activity (measured as number of steps per week) occurred through 18 months only for participants in the lowest two quartiles of physical activity at baseline. Participants in the highest quartile at baseline actually experienced a significant decrease in exercise through 18 months. Alcohol consumption significantly decreased only for participants in the highest quartile of alcohol consumption at baseline. Mean decrease in dietary cholesterol intake was significantly lower for participants in the first quartile of dietary cholesterol at baseline (−17) vs the forth quartile of dietary cholesterol at baseline (−178).

Using logistic regression, we found that the odds of improving physical or dietary behaviors from baseline through 18 months were significantly associated with the baseline quartile grouping for every variable except vegetable servings per day, whole grain servings per day, daily dietary cholesterol intake, and daily fiber intake. Age, sex, race, marital status, annual family income, educational status, and employment status were not associated with any of the physical or dietary behavior variables through 6 weeks or through 18 months, after adjusting for baseline quartile groupings (data not shown).

## Discussion

CHIP has demonstrated that a theory-based intervention conducted in a community setting can significantly improve most nutrition and physical activity behaviors for up to 18 months. The most dramatic improvements in both physical activity and nutrition behaviors occurred at 6 weeks. Because the most intensive part of the intervention ended after 4 weeks, it is possible that the largest improvement in behavior could have occurred somewhere after the baseline measure but before the 6-week measure. Depending on the behavior, health behavior decay does occur through 18 months, but most participants' variables did not return to baseline levels, especially for participants who started the program with higher risk behaviors.

In an ideal intervention, all behavior change patterns would appear like the data for total steps for participants in the first quartile. This group of participants averaged 19,536 steps per week at baseline. Individuals taking fewer than 35,000 steps per week are considered sedentary (26). Eighty-three percent of the participants in this group increased their physical activity through 6 weeks, and 87% increased their physical activity through 18 months. Hence, no health behavior decay was found for physical activity. At 18 months, participants in the first group are considered to be mostly sedentary, but they are twice as active as they were at baseline. Future research is needed to determine if this level of physical activity can produce meaningful health benefits.

The findings from CHIP are similar to those reported in the 18-month results of the PREMIER trial (27). The PREMIER trial compared the effects of three lifestyle interventions to treat prehypertension. In the trial, physical activity measures increased slightly at 6 and 18 months, although most of the nutrition variables spiked at 6 months, followed by a 1-year period of decline. Using a 6-month lifestyle change intervention to reduce cardiovascular risk factors in obese, sedentary, postmenopausal women, Carels et al demonstrated 6- and 12-month changes in physical activity with no decay, but newly adopted nutrition behaviors were not maintained and 63% of lost weight was regained at 12 months (28). The following year, Riebe et al showed that long-term maintenance of exercise and healthy eating behaviors in overweight adults can result in weight loss and maintenance of a healthy weight, as long as nutrition and physical activity behaviors are maintained (29).

Although some decay is expected, it can be overshadowed by significant improvements over baseline levels. For example, the participants in CHIP averaged 2.6 servings of fruits and vegetables per day at baseline. This jumped to four servings at 6 weeks and declined to 3.2 after 18 months. An improvement of one half of one serving after 18 months is both substantial and meaningful, even though the improvement is not as good as it was at 6 weeks.

The use of baseline quartiles to quantify behavior change over time is arbitrary, but it does have several advantages to a group mean. The average 18-month increase in fruit and vegetable servings was experienced by participants who needed to increase the most. From baseline to 18 months, the third and fourth quartiles for fruit servings and vegetable servings did not improve. These two quartiles were already averaging five servings of fruit and vegetables per day. Most of the improvements in fruit and vegetable consumption at 18 months were experienced by participants in the lowest two quartiles, who experienced three- and two-fold increases in servings. Documentation of improvements where they were most needed was evident for other nutrition variables (i.e., calories from fat, sweets, meat, dietary cholesterol, and saturated fat).

The analysis by quartiles also sheds additional light on the physical activity results. Participants in the highest quartile for physical activity at baseline were already taking approximately 11,000 steps per day (77,048 steps per week) and would not be expected to have much increase in physical activity. In this group, total steps actually decreased significantly.

Changing human behavior is one of the most daunting challenges for health professionals, yet it is the most important factor in chronic disease prevention. Some researchers have suggested that, because behavior change is so difficult, the most efficient way to improve health risks among the general population is to ensure healthy behaviors and attitudes are learned early in life (30). Nevertheless, even with an average age of 50 years, participants in CHIP were able to make significant improvements in healthy behaviors for at least 18 months. Although our current sociocultural-environmental realities make it difficult for people to change behaviors, helping adults make long-term behavior change still should be an important part of health risk reduction.

Wide-scale adoption and maintenance of healthy behaviors is difficult because, in the process of maintaining healthy behaviors, individuals experience changes in the determinants of healthy behavior. Armitage and Conner suggest that motivation, weighing of pros and cons, social influences, personal norms, and perceived behavioral control explain up to 50% of nutrition behavior (16). After the adoption of healthy behaviors, previous behaviors are not extinguished but go dormant, waiting for the right time and behavioral context to reemerge as the dominant behavior pattern (30). The interventions that address each of these determinants will stand the greatest chance of establishing long-term, healthy-behavior patterns. There are needs for a more advanced model of the maintenance process (i.e., one that views maintenance more as a journey than as a destination) and effective interventions that integrate individual-level policy influences with broader environmental- and macro-level policy influences (31).

The participants in this study were sufficiently self-motivated to volunteer to participate in a lifestyle change intervention. They were mostly white and slightly more educated than the community as a whole. Participants had lifestyles that permitted them to attend most, if not all, of the classes, evidenced by the high rate of attendance to this time-intensive program. The reminder phone call to participants to keep track of their daily pedometer data for that week may have caused an increase in physical activity beyond what participants were doing before the reminder. These limitations challenge the generalizability of the findings and make application of the intervention to other populations problematic. Because the study used an intensive lifestyle change program with participants who self-selected into the program, the results may represent a best-case scenario. Without a control group, determining how much of these improvements can be attributed to CHIP is not possible.

During an 18-month period, participants in CHIP demonstrated significant improvements in both nutrition and physical activity behaviors. With the exception of physical activity, the biggest improvements in behavior occurred at 6 weeks, but most nutrition behaviors still significantly improved after 18 months. These results are encouraging and consistent with other lifestyle trials. To improve the health and well-being of our population, greater adoption of health interventions such as CHIP appear warranted.

## Figures and Tables

**Table 1:** Bivariate Analyses at Baseline of Distribution of Variables by Whether Participants^a^ Remained in the Study Through 18 Months or Dropped Out, Coronary Health Improvement Project (CHIP), Rockford, Illinois, March 2003–August 2004

Characteristic	No. (%) Participants Who Remained in Study Through 18 Months (n = 211)	No. (%) Participants Who Dropped Out of Study (n = 137)	*Χ* ^2^	*P* Value
**Sex**				
Male	64 (30.3)	34 (24.8)	1.2	.26
Female	147 (69.7)	103 (75.2)		
**Race**				
White	197 (93.8)	130 (95.6)	0.5	.48
Other	13 (6.2)	6 (4.4)		
**Marital status**				
Never married	15 (7.2)	16 (11.9)	2.5	.47
Married	162 (77.9)	99 (73.9)		
Divorced	22 (10.6)	12 (9.0)		
Widowed	9 (4.3)	7 (5.2)		
**Employed**				
Yes	171 (81.0)	107 (81.7)	0	.88
No	40 (19.0)	24 (18.3)		
**Annual family income, $**				
0-20,000	16 (7.8)	10 (7.4)	0.8	.98
20,001-40,000	36 (17.6)	26 (19.3)		
40,001-60,000	47 (22.9)	31 (22.9)		
>60,000	106 (51.7)	68 (50.4)		
**Educational status**				
<High school	8 (3.8)	3 (2.2)	3.0	.56
High school degree	48 (23.0)	35 (25.7)		
Some college	56 (26.8)	41 (30.2)		
College degree	52 (24.9)	25 (18.4)		
Post college degree	45 (21.5)	32 (23.5)		

a Because of missing data, the number of participants who remained in the study may not total to 211, and the number of participants who dropped out of the study may not total to 137.

**Table 2:** Mean Change in Selected Physical and Dietary Behaviors From Baseline Through 6 Weeks and Through 18 Months (N = 211), Coronary Health Improvement Project (CHIP), Rockford, Illinois, March 2003–August 2004

Physical and Dietary Behavior	Mean at Base-line	Mean Change Through 6 Weeks (95% CI)	% of Participants Who Improved Over 6 Weeks	Mean Change Through 18 Months (95% CI)	% of Participants Who Improved Over 18 Months
Physical activity, no. steps/week	47,438	7,436 (4,913 to 9,958)	66	5,596 (2,360 to 8,832)	63
No. kcal/day	1,894	−339 (−437 to −242)	71	−391 (−473 to −310)	75
% kcal from fat/day	36	−9 (−10 to −7)	84	−4 (−5 to −3)	74
% kcal from protein/day	15	−0.8 (−1.2 to −0.4)	62	−0.3 (−0.7 to 0)	53
% kcal from carbohydrates/day	231	2.4 (−9.0 to 13.9)	49	−27 (−38 to −16)	64
% kcal from sweets/day	14	−6.8 (−8.1 to −5.4)	80	−3 (−4 to −2)	69
% kcal from alcohol/day	2.5	−1.0 (−1.3 to −0.6)	53	−0.2 (−0.6 to 0.1)	42
Fiber from fruits and vegetables, g/day	8.2	6.0 (5.2 to 6.8)	83	2.6 (1.8 to 3.3)	67
Vegetables, no. servings/day	3.6	1.9 (1.5 to 2.2)	78	0.7 (0.4 to 1.1)	59
Fruit, no. servings/day	1.6	1.1 (0.9 to 1.3)	76	0.4 (0.3 to 0.6)	58
Whole grains, no. servings/day	5.0	1.5 (1.2 to 1.9)	70	−0.1 (−0.4 to 0.2)	47
Meat, no. servings/day	1.9	−0.5 (−0.7 to −0.3)	72	−0.5 (−0.6 to −0.4)	73
Dairy, no. servings/day	1.3	−0.8 (−0.9 to −0.6)	80	−0.6 (−0.8 to −0.5)	74
Fat, no. servings/day	2.8	−1.5 (−1.7 to −1.3)	84	−1.0 (−1.2 to −0.8)	78
Total dietary fat, g/day	77	−29 (−34 to −23)	83	−23 (−27 to −19)	78
Dietary cholesterol, mg/day	190	−103 (−121 to −85)	91	−80 (−95 to −65)	84
Polyunsaturated fat, g/day	19	−6 (−7 to −4)	75	−5 (−6 to −4)	73
Monounsaturated fat, g/day	30	−12 (−14 to −9)	83	−9 (−11 to −7)	76
Saturated fat, g/day	22	−10 (−12 to −8)	87	−8 (−9 to −7)	83
Protein, g/day	72	−15 (−19 to −11)	75	−16 (−19 to −12)	75
Fiber, g/day	20	10 (8 to 12)	79	4 (2 to 5)	67

CI indicates confidence interval.

**Table 3:** Mean Change in Selected Physical and Dietary Behaviors From Baseline Through 6 Weeks and Through 18 Months, According to Quartile Groupings at Baseline (N = 211), Coronary Health Improvement Project (CHIP), Rockford, Illinois, March 2003–August 2004

Quartile (No. Participants at Baseline)	Mean at Baseline	**Mean** a ** Change Through 6 Weeks (95% CI)**	% Participants Who Improved Over 6 Weeks	**Mean** a ** Change Through 18 Months (95% CI)**	% Participants Who Improved Over 18 Months
**Physical activity (no. steps/week)^b^**					
Quartile 1 (52)	19,536	14,069 (a) (9,105 to 19,032)	83	17,933 (a) (13,344 to 22,523)	87
Quartile 2 (53)	38,851	14,362 (a) (8,151 to 20,573)	77	11,532 (a) (4,895 to 18,169)	64
Quartile 3 (52)	53,913	4,587 (b) (1,160 to 8,013)	62	1,952 (b) (−3,419 to 7,323)	60
Quartile 4 (53)	77,048	−3,202 (c) (−7,074 to 670)	43	−8,412 (c) (−15,470 to −1,354)	36
**No. kcal/day**					
Quartile 1 (53)	1,075	94 (a) (−26 to 213)	47	−58 (a) (−155 to 38)	62
Quartile 2 (53)	1,532	−92 (a) (−218 to 34)	64	−239 (a, b) (−348 to −129)	77
Quartile 3 (52)	1,997	−327 (b) (−469 to −18)	77	−377 (b) (−528 to −226)	71
Quartile 4 (53)	2,958	−1,014 (c) (−1,244 to −786)	94	−890 (c) (−1,083 to −697)	91
**% kcal from fat/day**					
Quartile 1 (53)	26	−3 (a) (−5 to −2)	75	−0.4 (a) (−1.9 to 1.1)	58
Quartile 2 (52)	34	−7 (b) (−9 to −5)	87	−3 (b) (−5 to −2)	77
Quartile 3 (53)	38	−8 (b) (−10 to −6)	81	−4 (b) (−6 to −2)	72
Quartile 4 (53)	46	−16 (c) (−17 to −13)	94	−9 (c) (−11 to −7)	89
**% kcal from protein/day**					
Quartile 1 (52)	12	1.1 (a) (0 to 2)	35	1.6 (a) (1 to 3)	17
Quartile 2 (53)	14	0 (b) (−1 to 1)	42	0.1 (b) (0 to 1)	51
Quartile 3 (53)	16	−0.8 (b) (−1 to 0)	72	−0.3 (b) (−1 to 0)	53
Quartile 4 (53)	19	−3.4 (c) (−4 to −3)	98	−2.7 (c) (−4 to −2)	83
**% kcal from carbohydrates/day**					
Quartile 1 (52)	132	46 (a) (28 to 63)	26	0 (a) (−16 to 16)	57
Quartile 2 (54)	188	29 (a, b) (12 to 47)	36	−5 (a) (−19 to 10)	55
Quartile 3 (52)	248	9 (b) (−10 to 28)	53	−25 (a) (−44 to −6)	64
Quartile 4 (53)	358	−74 (c) (−98 to −51)	83	−79 (b) (−107 to −51)	79
**% kcal from sweets/day**					
Quartile 1 (53)	4	−0.4 (a) (−1.5 to 0.7)	60	1.7 (a) (0.6 to 2.8)	43
Quartile 2 (51)	9	−3 (b) (−5 to −2)	84	−2 (b) (−3 to −1)	73
Quartile 3 (54)	15	−6 (c) (−8 to −5)	80	−4 (b) (−6 to −2)	76
Quartile 4 (53)	28	−16 (d) (−19 to −13)	94	−9 (c) (−12 to −5)	85
**% kcal from alcohol/day**					
Quartile 1 (50)	0	0 (a) (0)	NA	0 (a) (0)	NA
Quartile 2 (56)	0.2	−0.1 (a) (−0.2 to 0)	52	0.1 (a) (−0.1, to 0.2)	43
Quartile 3 (52)	1.3	−0.3 (a) (−0.7 to 0.1)	75	0.2 (a) (−0.1 to 0.4)	50
Quartile 4 (53)	8.2	−3.5 (b) (−4.7 to −2.3)	81	−1.3 (b) (−2.7 to 0)	72
**Fiber from fruits and vegetables, g/day**					
Quartile 1 (52)	3.6	6.8 (a) (5.4 to 8.1)	94	3.7 (a) (2.2 to 5.2)	79
Quartile 2 (53)	5.9	6.4 (a) (4.7 to 8.0)	87	3.3 (a) (2.1 to 4.5)	75
Quartile 3 (54)	8.5	6.6 (a) (4.9 to 8.3)	85	2.0 (a) (0.5 to 3.5)	56
Quartile 4 (52)	14.8	4.1 (a) (2.5 to 5.7)	67	1.3 (a) (−0.4 to 3.0)	58
**Vegetables, no. servings/day**					
Quartile 1 (51)	1.5	2.3 (a) (1.7 to 2.9)	94	1.4 (a) (0.7 to 2.1)	71
Quartile 2 (54)	2.5	2.0 (a) (1.3 to 2.6)	78	1.0 (a, b) (0.4 to 1.6)	59
Quartile 3 (53)	3.7	2.1 (a) (1.4 to 2.8)	81	0.7 (a, b) (0.1 to 1.3)	53
Quartile 4 (53)	6.7	1.2 (a) (0.3 to 2.0)	60	−0.2 (b) (−1.1 to 0.7)	53
**Fruits, no. servings/day**					
Quartile 1 (49)	0.4	1.6 (a) (1.2 to 1.9)	82	0.7 (a) (0.4 to 0.9)	76
Quartile 2 (46)	0.9	1.6 (a) (1.2 to 1.9)	89	0.8 (a) (0.5 to 1.0)	76
Quartile 3 (60)	1.6	1.2 (a) (0.9 to 1.5)	82	0.4 (a) (0.1 to 0.7)	52
Quartile 4 (56)	3.2	0.2 (b) (−0.1 to 0.5)	54	−0.1 (b) (−0.4 to 0.3)	38
**Whole grains, no. servings/day**					
Quartile 1 (53)	2.2	2.4 (a) (1.7 to 3.1)	87	0.8 (a) (0.2 to 1.3)	57
Quartile 2 (51)	3.8	2.0 (a) (1.3 to 2.7)	78	0.3 (a) (−0.2 to 0.9)	55
Quartile 3 (53)	5.4	1.5 (a) (0.8 to 2.3)	64	−0.1 (a) (−0.8 to 0.6)	43
Quartile 4 (54)	8.3	0.4 (b) (−0.4 to 1.1)	50	−1.3 (b) (−2.1 to −0.5)	35
**Meat, no. servings/day**					
Quartile 1 (46)	0.8	0.1 (a) (−0.1 to 0.3)	54	0.1 (a) (0 to 0.3)	46
Quartile 2 (52)	1.3	−0.2 (a, b) (−0.4 to −0.1)	67	−0.2 (b) (−0.4 to −0.1)	67
Quartile 3 (58)	2.0	−0.5 (b) (−0.7 to −0.2)	78	−0.5 (b) (−0.7 to −0.3)	79
Quartile 4 (55)	3.5	−1.4 (c) (−1.8 to −1.0)	85	−1.3 (c) (−1.6 to −1.0)	93
**Dairy, no. servings/day**					
Quartile 1 (52)	0.3	−0.1 (a) (−0.2 to 0)	54	0.1 (a) (0 to 0.1)	50
Quartile 2 (49)	0.7	−0.4 (b) (−0.5 to −0.3)	86	−0.2 (a) (−0.4 to −0.1)	71
Quartile 3 (54)	1.3	−0.7 (b) (−0.9 to −0.5)	83	−0.6 (b) (−0.8 to −0.4)	83
Quartile 4 (56)	2.8	−1.8 (c) (−2.2 to −1.5)	95	−1.6 (c) (−2.0 to −1.3)	89
**Fat, no. servings/day**					
Quartile 1 (48)	1.0	−0.2 (a) (−0.4 to 0.1)	65	0 (a) (−0.3 to 0.2)	63
Quartile 2 (52)	2.0	−0.9 (b) (−1.2 to −0.6)	83	−0.4 (a) (−0.6 to −0.1)	65
Quartile 3 (57)	3.1	−1.7 (c) (−2.0 to −1.4)	93	−1.3 (b) (−1.6 to −0.9)	89
Quartile 4 (54)	5.0	−2.9 (d) (−3.4 to −2.5)	94	−2.2 (c) (−2.7 to −1.8)	93
**Total dietary fat, g/day**					
Quartile 1 (53)	36	−5 (a) (−10 to −0.1)	70	−6 (a) (−9 to −3)	64
Quartile 2 (53)	59	−12 (a) (−19 to −6)	83	−9 (a) (−15 to −2)	68
Quartile 3 (52)	82	−25 (b) (−34 to −15)	81	−24 (b) (−30 to −18)	88
Quartile 4 (53)	132	−72 (c) (−83 to −60)	100	−53 (c) (−63 to −43)	92
**Dietary cholesterol, mg/day**					
Quartile 1 (53)	71	−24 (a) (−41 to −7)	92	−17 (a) (−29 to −6)	75
Quartile 2 (52)	129	−61 (a) (−75 to −46)	87	−45 (a, b) (−58 to −31)	83
Quartile 3 (53)	197	−104 (b) (−135 to −74)	92	−78 (b) (−96 to −60)	89
Quartile 4 (53)	358	−223 (c) (−269 to −177)	94	−178 (c) (−221 to −134)	89
**Polyunsaturated fat, g/day**					
Quartile 1 (53)	8	2 (a) (0.2 to 3.7)	45	0.2 (a) (−0.9 to 1.3)	53
Quartile 2 (52)	14	−3 (b) (−4 to −1)	77	−1 (a) (−3 to 0.1)	63
Quartile 3 (53)	20	−5 (b) (−7 to −3)	81	−5 (b) (−7 to −3)	83
Quartile 4 (53)	33	−16 (c) (−19 to −13)	98	−13 (c) (−15 to −11)	94
**Monounsaturated fat, g/day**					
Quartile 1 (53)	13	−2 (a) (−4 to 1)	66	−1 (a) (−3 to 1)	6
Quartile 2 (52)	22	−7 (b) (−9 to −5)	90	−4 (a) (−6 to −2)	71
Quartile 3 (53)	32	−9 (b) (−13 to −5)	79	−9 (b) (−11 to −6)	79
Quartile 4 (53)	52	−28 (c) (−32 to −23)	98	−21 (c) (−25 to −17)	91
**Saturated fat, g/day**					
Quartile 1 (53)	10	−2 (a) (−3 to 0)	72	−2 (a) (−3 to −1)	66
Quartile 2 (52)	16	−6 (b) (−7 to −4)	87	−5 (a) (−6 to −3)	81
Quartile 3 (53)	23	−10 (c) (−13 to −8)	91	−8 (b) (−10 to −6)	91
Quartile 4 (53)	40	−23 (d) (−27 to −18)	98	−17 (c) (−21 to −14)	92
**Protein, g/day**					
Quartile 1 (52)	38	2 (a) (−3 to 8)	60	−3 (a) (−6 to 1)	58
Quartile 2 (50)	56	−5 (a, b) (−10 to −1)	70	−7 (a) (−12 to −3)	68
Quartile 3 (58)	77	−14 (b) (−19 to −9)	74	−16 (b) (−21 to −10)	79
Quartile 4 (51)	114	−44 (c) (−53 to −35)	96	−37 (c) (−45 to −29)	94
**Fiber, g/day**					
Quartile 1 (53)	10	12 (a) (9 to 15)	85	6 (a) (3 to 8)	77
Quartile 2 (53)	15	12 (a) (9 to 14)	91	3 (a) (1 to 5)	68
Quartile 3 (51)	21	13 (a) (9 to 17)	76	7 (a, b) (3 to 10)	65
Quartile 4 (54)	34	4 (b) (1 to 10)	63	0.4 (b) (−3 to 4)	57

NA indicates not applicable; CI, confidence interval.

a The Student-Newman-Keuls test for multiple comparisons was used to compare means at baseline, 6 weeks, and 18 months and to compare means among the quartile groups. Means with the same letters are not significantly different.

b Data are missing for one participant in this category.

## References

[1] Bellg AJ (2003). Maintenance of health behavior change in preventive cardiology. Internalization and self-regulation of new behaviors. Behav Modif.

[2] Marcus BH, Dubbert PM, Forsyth LH, McKenzie TL, Stone EJ, Dunn AL, Blair SN (2000). Physical activity behavior change: issues in adoption and maintenance. Health Psychol.

[3] Anderson JW, Konz EC, Frederich RC, Wood CL (2001). Long-term weight-loss maintenance: a meta-analysis of US studies. Am J Clin Nutr.

[4] Fogelholm M, Kukkonen-Harjula K (2000). Does physical activity prevent weight gain — a systematic review. Obes Rev.

[5] Dusing R (2006). Overcoming barriers to effective blood pressure control in patients with hypertension. Curr Med Res Opin.

[6] Miura K, Nakagawa H (2005). Can dietary changes reduce blood pressure in the long term?. Curr Opin Nephrol Hypertens.

[7] Howard BV, Van Horn, Hsia J, Manson JE, Stefanick ML, Wassertheil-Smoller S (2006). Low-fat dietary pattern and risk of cardiovascular disease: the Women's Health Initiative Randomized Controlled Dietary Modification Trial. JAMA.

[8] Pritchett AM, Foreyt JP, Mann DL (2005). Treatment of the metabolic syndrome: the impact of lifestyle modification. Curr Atheroscler Rep.

[9] Haynes RB, McDonald HP, Garg AX (2002). Helping patients follow prescribed treatment: clinical applications. JAMA.

[10] Englert HS, Diehl HA, Greenlaw RL (2004). Rationale and design of the Rockford CHIP, a community-based coronary risk reduction program: results of a pilot phase. Prev Med.

[11] Diehl HA (1998). Coronary risk reduction through intensive community-based lifestyle intervention: the Coronary Health Improvement Project (CHIP) experience. Am J Cardiol.

[12] Aldana SG, Greenlaw RL, Diehl HA, Salberg A, Merrill RM, Ohmine S (2005). Effects of an intensive diet and physical activity modification program on the health risks of adults. J Am Diet Assoc.

[13] Aldana SG, Greenlaw RL, Diehl HA, Salberg A, Merrill RM, Ohmine S (2006). The behavioral and clinical effects of therapeutic lifestyle change on middle-aged adults. Prev Chronic Dis.

[14] Green LW, Kreuter NW (1999). Health promotion planning: an educational and ecological approach.

[15] McKenzie JF, Smeltzer JL (2001). Planning, implementing, and evaluating health promotion programs: a primer.

[16] Armitage CJ, Conner M (2000). Social cognition models and health behaviour: a structured review. Psychol Health.

[17] Block G, Woods M, Potosky A, Clifford C (1990). Validation of a self-administered diet history questionnaire using multiple diet records. J Clin Epidemiol.

[18] Block G, Thompson FE, Hartman AM, Larkin FA, Guire KE (1992). Comparison of two dietary questionnaires validated against multiple dietary records collected during a 1-year period. J Am Diet Assoc.

[19] Mares-Perlman JA, Klein BE, Klein R, Ritter LL, Fisher MR, Freudenheim JL (1993). A diet history questionnaire ranks nutrient intakes in middle-aged and older men and women similarly to multiple food records. J Nutr.

[20] Tudor-Locke C, Williams JE, Reis JP, Pluto D (2004). Utility of pedometers for assessing physical activity: construct validity. Sports Med.

[21] Crouter SE, Schneider PL, Karabulut M, Bassett DR (2003). Validity of 10 electronic pedometers for measuring steps, distance, and energy cost. Med Sci Sports Exerc.

[22] Tudor-Locke C, Williams JE, Reis JP, Pluto D (2002). Utility of pedometers for assessing physical activity: convergent validity. Sports Med.

[23] Fienberg SE (1977). The analysis of cross-classified data.

[24] Mantel N, Haenszel W (1959). Statistical aspects of the analysis of data from retrospective studies of disease. J Natl Cancer Inst.

[25] Everitt BS, Dunn G (1991). Applied multivariate data analysis.

[26] Tudor-Locke C, Bassett DR (2004). How many steps/day are enough? Preliminary pedometer indices for public health. Sports Med.

[27] Elmer PJ, Obarzanek E, Vollmer WM, Simons-Morton D, Stevens VJ, Young DR (2006). Effects of comprehensive lifestyle modification on diet, weight, physical fitness, and blood pressure control: 18-month results of a randomized trial. Ann Intern Med.

[28] Carels RA, Darby LA, Cacciapaglia HM, Douglass OM (2004). Reducing cardiovascular risk factors in postmenopausal women through a lifestyle change intervention. J Womens Health (Larchmt).

[29] Riebe D, Blissmer B, Greene G, Caldwell M, Ruggiero L, Stillwell KM (2005). Long-term maintenance of exercise and healthy eating behaviors in overweight adults. Prev Med.

[30] Bouton ME (2000). A learning theory perspective on lapse, relapse, and the maintenance of behavior change. Health Psychol.

[31] Orleans CT (2000). Promoting the maintenance of health behavior change: recommendations for the next generation of research and practice. Health Psychol.

